# Getting the whole story: Integrating patient complaints and staff reports of unsafe care

**DOI:** 10.1177/13558196211029323

**Published:** 2021-07-07

**Authors:** Jackie Van Dael, Alex Gillespie, Tom Reader, Katelyn Smalley, Dimitri Papadimitriou, Ben Glampson, Daniel Marshall, Erik Mayer

**Affiliations:** 1 Research Associate, NIHR Imperial Patient Safety Translational Research Centre, Institute of Global Health Innovation, 4615 Imperial College London, UK; 2 Associate Professor, Department of Psychological and Behavioural Science, London School of Economics, UK; 3 PhD Candidate, NIHR Imperial Patient Safety Translational Research Centre, Institute of Global Health Innovation, 4615 Imperial College London, UK; 4 Deputy Research Informatics Programme Manager, Imperial College Healthcare NHS Trust, London, UK; 5 Research Informatics Programme Manager, Imperial College Healthcare NHS Trust, London, UK; 6 Complaints and Service Improvement Manager, Imperial College Healthcare NHS Trust, London, UK; 7 Clinical Senior Lecturer, NIHR Imperial Patient Safety Translational Research Centre, Institute of Global Health Innovation, 4615 Imperial College London, UK

**Keywords:** patient involvement, complaints, incident reporting systems

## Abstract

**Objective:**

It is increasingly recognized that patient safety requires heterogeneous insights from a range of stakeholders, yet incident reporting systems in health care still primarily rely on staff perspectives. This paper examines the potential of combining insights from patient complaints and staff incident reports for a more comprehensive understanding of the causes and severity of harm.

**Methods:**

Using five years of patient complaints and staff incident reporting data at a large multi-site hospital in London (in the United Kingdom), this study conducted retrospective patient-level data linkage to identify overlapping reports. Using a combination of quantitative coding and in-depth qualitative analysis, we then compared level of harm reported, identified descriptions of adjacent events missed by the other party and examined combined narratives of mutually identified events.

**Results:**

Incidents where complaints and incident reports overlapped (n = 446, reported in 7.6%’ of all complaints and 0.6% of all incident reports) represented a small but critical area of investigation, with significantly higher rates of Serious Incidents and severe harm. Linked complaints described greater harm from safety incidents in 60% of cases, reported many surrounding safety events missed by staff (n = 582), and provided contesting stories of why problems occurred in 46% cases, and complementary accounts in 26% cases.

**Conclusions:**

This study demonstrates the value of using patient complaints to supplement, test, and challenge staff reports, including to provide greater insight on the many potential factors that may give rise to unsafe care. Accordingly, we propose that a more holistic analysis of critical safety incidents can be achieved through combining heterogeneous data from different viewpoints, such as through the integration of patient complaints and staff incident reporting data.

## Introduction

With around one in 20 patients experiencing preventable harm,^1^ patient safety has become one of the biggest global health challenges of our time. Several databases and incident reporting systems have been established based on the premise that staff reports of incidents and near-misses can help identify and mitigate future risks. Yet, despite their widespread use, cost, and a wealth of consequent data, their impact on improving safety has remained unclear.^2,3^

Contrary to the ambition of transforming incident reporting systems into epidemiological models of adverse events, it is becoming increasingly clear that reported incidents often reflect trends in cultures of learning and reporting, rather than actual patterns of unsafe care.^4^ The insights that staff provide on the causes and severity of incidents rely on a range of factors, including a hospital’s safety culture, a staff member’s personal sense-making of whether poor outcomes are avoidable and classify as reportable incidents, and the purposes and structure of their respective mechanisms for reporting. Due to these considerations, the importance of supplementing, testing, and challenging internal understandings of safety within health care organizations has been suggested.^5^ In this paper, we propose that increased linkage of heterogeneous datasets provided by different stakeholders, and in particular patients and staff, may be an important source of insight towards this end.

### The potential value of combining patient and staff datasets for patient safety

Patient-generated data can be considered a valuable source of safety insight for a number of reasons. Patients are independent from a hospital’s norms and culture, meaning they are not influenced by safety culture limitations that shape staff reporting (e.g. fear of blame),^6–8^ and can detect problems when internal systems fail (e.g. such as reflected in the Mid-Staffordshire Inquiry).^9^ Further, while staff’s sense-making of safety is shaped by professional practice and often focuses on biomedical explanations for harm, patients tend to report on socio-structural factors (e.g. institutional processes, missed concerns), which are known contributory factors to adverse events, and critical for the improvement of systems for safer care.^10–12^ Unlike staff (who are limited by their episodic interactions with patients), patients are also able to identify problems across space and over time, including issues across visits and outside of organizational boundaries (e.g. care continuity breakdowns, failed care coordination).^13^ Finally, while staff are limited by the structured and predefined nature of incident reporting systems,^14^ patients can provide complex stories of the pathway to harm, including how problems might inter-relate and unfold over time.

These differences suggest that combining patient and staff datasets on safety may have two important contributions. First, by combining heterogeneous data generated by different stakeholders (i.e. patients and staff), differential aspects of unsafe care may be highlighted, and contribute to a more comprehensive view of the many social, clinical, psychological factors that give rise to unsafe care. Second, analysing the patient’s viewpoint can provide a frame of reference to interpret staff narratives of unsafe care, which can challenge internal assumptions about safety or highlight informational gaps in datasets generated from singular viewpoints.

### Current study and theoretical underpinnings

In this study, we link and combine patient complaints and staff incident reports, through retrospective patient-level data linkage at a multisite hospital in London, UK. Complaints were used because they are a valid (e.g. in terms of associations with incident reports, hospital mortality rates and adverse surgical outcomes),^15–17^ but often neglected,^18^ patient-initiated safety data source, in which patients provide narrative accounts of specific adverse events and near-misses.

Combining insights from staff incident reports and patient complaints may be valuable for several reasons. First, incidents captured in both systems represent important instances of failure (staff and patient regarded the incidents as reaching the threshold for reporting), and potential points of rupture between perspectives (as indicated by the patient’s act of complaining). Second, each report is situated in a different ‘learning’ or governance systems, meaning reporting purposes may vary across the reporting communities (e.g. avoiding blame versus seeking redress), which may have implications on how the story is told from each point of view. Third, there are differences in the spatio-temporal conditions for reporting across the two systems (short-term report versus retrospective reflection), meaning the episodic and decontextualized nature of incident reports may be supplemented by the patient journey as described in the linked complaint.^14^

Drawing from existing evidence on the insights held within complaints and incident reports, we explored the combined reports through three research aims.

### Comparing patient and staff reports of harm

We first compare patient and staff reports of patient harm resulting from mutually identified events. Existing research on the content of overlap between patient complaints and staff incident reports is limited, and it is unknown whether patients and staff provide similar information on level of harm (a commonly used indicator for deeper investigation) resulting from a mutually identified incident.

### Examining adjacent events

We then examine patient versus staff reports of adjacent events. When describing the care period surrounding a mutually identified event, patient and staff might identify surrounding events that contributed to harm but were missed by the other party. Patient complaints regularly refer to multiple distinct but related events in their journeys of care,^13^ whereas staff incident reports describe events in isolation – a function of their episodic interaction with individual patients.^7^ This means patients can report on important determinants of harm that staff are unable to account for, including events that are invisible to staff (that is, events that occur outside organizational boundaries). Similarly, staff can also observe and report additional events surrounding mutually identified events that patients did not witness (e.g. internal systems issues). We refer to these as ‘adjacent events’ and explore to what extent patient and staff report these and how critical they are in terms of subsequent harm.

### Combining descriptions of mutually identified events

We finally explore the supplementary value of integrating patient and staff reports of mutually identified events. When patients and staff describe the same event (what we term ‘mutually identified events’), they may provide divergent descriptions of how incidents were caused due to discrepant situation and role demands. For example, staff may be tended to limit disclosure due to a hospital’s poor safety culture (e.g. fear of blame),^8^ or omit difficult-to-identify problems (e.g. omissions, patient misunderstanding). In contrast, patient accounts are independent from a hospital’s norms and culture, and their complaints often highlight complex social and systems factors,^11^ but could also underestimate inherent procedural risk factors, or blame staff unjustly (e.g. racist complaints).

## Methods

### Study setting

This study was conducted at a large multi-site teaching hospital in England. This setting includes five acute sites and a range of community services. The hospital is one of the largest in the country with respect to health care provision (approximately 1.5 million patients per year), with a yearly average of 1,134 complaints in the past five years, and an average incident reporting rate of 45.6 per 1,000 bed days between October 2018 and April 2019. 

### Data characteristics and preparation

Table 1 presents the key characteristics of staff incident reports, named ‘Patient Safety Incidents’ (PSIs) in the English National Health Service (NHS), and patient complaints. For each patient with a recorded complaint between April 2014 and March 2019 (n = 5,265), PSI records were searched to identify incidents reported by staff. Full details of these datasets and how they were linked can be found in the Online Supplement 1.

**Table 1.** Key characteristics of datasets used in this study.

	Definition	Primary function for patient safety
Patient Safety Incident (PSI)	A short report typically submitted by frontline staff to record ‘any unintended or unexpected incident which could have, or did, lead to harm for one or more patients receiving healthcare.’^19^^( p.2)^	1. *Local*: To trigger investigations into critical incidents within a care setting (e.g. when a PSI has resulted in significant harm or is considered a Serious Incident). 2. *National*: To monitor incidents across institutions and inform national priority setting.
Patient complaint	A complex narrative submitted by patients and families to report on perceived failures of healthcare delivery – primarily to drive quality improvement and seek answers.^18^	1. *Local*: Complaints are formally investigated by the recipient hospital to identify whether reported concerns were well-founded and require action. 2. *National*: To monitor problem themes and service areas reported in complaints across institutions

Linked complaints were operationalized as all complaints that described at least one incident that directly matched a staff incident report (i.e. a mutually identified event) based on the incident date, location, and a manual content review of the incident description.

As complaints often report on multiple – and potentially inter-related - problems, we further included any descriptions of events before or after a mutually identified event that were captured in linked complaints (i.e. patient-reported adjacent events). Similarly, for staff incident reports, we also extracted any incidents within the same timeframe of a mutually identified event that were not captured in the linked complaint (i.e. staff-reported adjacent events).

In short, mutually identified events were operationalized as (perceived) errors or omissions reported by both the patient (in their complaint) and staff (in their incident report). Adjacent events were operationalized as (perceived) errors or omissions that occurred within the timeframe of a mutually identified event (as set out in the complaint), but were only reported by one party (i.e. in a staff incident report or in a patient’s complaint).

All mutually identified and adjacent events were extracted from complaint letters and the hospital data system for further coding and analysis.

### Coding framework

To enable aggregated comparisons between the datasets, patient and staff descriptions were codified using standardised protocols (Table 2).

**Table 2.** Classifications used for patient and staff reported events.

Code	Description
Reporter type	*Who reported this event?* Both (i.e. mutually identified event) Patient-only (i.e. an adjacent event reported only by the patient) Staff-only (i.e. an adjacent event reported only by staff)
Problem domain	*What does the patient or staff report describe as the overall problem domain of the event?* Clinical: Issues relating to the quality and safety of clinical and nursing care Management: Issues relating to the environment and organisation within which health care is provided Relationship: Issues relating to the behaviour of any member of staff towards patients or vice versa
Problem category	*What is the specific problem category reported by staff?* Blood transfusion; communication. diagnosis. diagnostics and investigations. discharge. implementation of care. infrastructure. labour and delivery. medical device or equipment. medication. operation or procedures. patient information. pressure ulcer. slips, trips, or falls. transfer
	*What is the specific problem category reported by the patient?* Quality; safety. environment. institutional. listening. communication. respect and rights
Level of harm^a^	*What is the level of harm reported in the patient or staff report as a result of the described event?* Near-miss: Potential for harm, but this was prevented No harm: No harm Low: Minimal harm requiring extra observation or minor treatment Moderate: Short-term harm requiring further treatment or surgery Major: Permanent or long-term harm Death: Patient death N/A: Insufficient information on harm is reported

^a^In complaints, harm was only recorded if an event was explicitly described as (at least partially) causing harm. In some cases, multiple events together led to a certain level of harm (e.g. both coded at the same level).

To examine whether patients and staff generally reported similar problem themes behind mutually identified events, free-text descriptions were categorized based on a high-level tri-partite distinction (clinical, management, relationship) derived from wider healthcare delivery theory and practice.^20^ These three domains were considered sufficiently broad and exhaustive to apply to both datasets (as confirmed by the inter-rater reliability results reported below).

To identify and map more detailed characteristics of patient versus staff descriptions, lower-level categories were used validated protocols specific to each dataset. Specifically, the existing English NHS’ National Reporting and Learning System’s problem categories were used for staff incident reports. The Healthcare Complaints Analysis Tool (HCAT),^20^ a psychometrically reliable and theoretically informed framework for analysing complaints, was used to codify problem categories as described in complaints.

The NRLS and HCAT taxonomies were also used to classify levels of reported harm for staff versus patient reports. The levels of harm in HCAT are derived from the NRLS’ defined levels of harm, meaning harm severity was directly comparable between the two datasets.

### Coding reliability

All patient and staff reports were analysed by the main author (JD). To evaluate coding reliability, 127 events, including 60 staff reports (10.5% of all staff reports) and 108 patient reports (10.5% of all patient reports), were also coded by a second health policy researcher (KS). Events were extracted from a randomly selected sample of 35 complaints (8.7% of all complaints) and coding was blinded between coders. Domain and problem category were tested using Gwet’s AC1 (which corrects for problem distribution). Harm was tested using weighted kappa. Reports with insufficient information on harm were coded as ‘n/a’ and excluded from the test. Near-misses were included as ‘no harm’.

Gwet’s AC1 for domains and problem categories applied to patient reports (n = 108) found AC1 = 0.69 (95% CI, 0.59 to 0.81) and AC1 = 0.55 (95% CI, 0.44 to 0.66), respectively. Weighted kappa for level of harm in patient reports (n = 101) found κ = 0.63, z = 9.98. Gwet’s AC1 for problem domains applied to staff reports (n = 60) indicated AC1 = 0.78 (95% CI, 0.64 to 0.91). Rater disagreement on whether events were correctly matched occurred in one out of 41 mutually identified events (2.4%).

### Analysis process

#### Characteristics of overlap between complaints and staff incident reports

To examine what types of staff incident reports were more likely to have an overlapping complaint, chi-square tests of independence were generated to test associations between the presence of a complaint and hospital-reported data on incident type, severity (e.g., Serious Incident or not), and level of harm (full details in Online Supplement 1).

#### Comparing patient and staff reports of harm

We then examined differences between levels of harm attributed to mutually identified events. The association between level of harm reported by staff versus the patient for mutually identified events was calculated with Spearman’s rho.

#### Examining adjacent events

Problem categories of adjacent events reported by patients versus reported by staff were examined based on frequencies of the codified problem categories. We further generated mosaic plots and chi-square tests of independence to explore associations between problem categories and resulting harm (reported in Online Supplement 2). Free-text reports of the sub-set of adjacent events that were associated with significant harm underwent thematic analysis^21^ to identify type of instances.

#### Combining descriptions of mutually identified events

We then compared patient and staff reports of mutually identified events to identify if, when, and how the patient and staff reports provide differential information on the same events. For each mutually identified event, we conducted a qualitative content analysis of the matched reports, which identified two main types of difference between patient and staff reports: those that were complementary (‘complementary accounts’) and those that were contesting (‘contested stories’). Each mutually identified event was then labelled according to either of these types of difference (with the exception of a minority of comparative reports that were neither). Following a thematic analysis approach,^22^ comparative descriptions of mutually identified events were then analysed in an iterative manner to develop sub-themes within each type of difference. To indicate for what sorts of incidents reports were contested versus complementary, we have also reported problem category proportions (as codified with the standardized protocols in Table 2) of patient and staff reports within each type of difference.

Quantitative analyses and figures were produced using R software. Qualitative analyses were performed in Excel.

## Results

A total of 446 events were reported by both patients (in 402 (7.6%) of all 5,265 complaints) and staff (in 0.6% of all 81,077 staff reports). The NHS defines a Serious Incident regarding PSIs as one ‘where the consequences to patients, families and carers, staff or organisations are so significant or the potential for learning is so great, that a heightened level of response is justified.’^23^^(p.7)^ The area of overlap between PSIs and complaints represented a critical area of investigation, with staff reports being 5.8 times more likely to be recorded as a Serious Incident by the hospital (compared to non-linked PSIs) (χ^2^ = 46.64, p < .001) (Online Supplement 1). Staff incident reports with a linked complaint were also significantly more likely to have been reported by staff as resulting in moderate (χ^2^ = 230.96, p < .001), major (χ^2^ = 66.42, p < .001), or catastrophic harm (χ^2^ = 169.91, p < .001) compared to reports without an overlapping patient complaint.

Of all 446 mutually identified events, 123 staff reports (27.6%) were instigated by patient concerns, indicating these might have otherwise been missed.

Of 909 complaints initially linked at the patient level, 507 complaints were excluded due to an absence of a matching incident (n = 363) (despite reporting on the same patient and care period), incorrect patient linkage (n = 107) (e.g. same name, different patient), missing or incomplete records (n = 21) and not being submitted by patients (n = 16) (e.g. clinician complaint).

### Patient versus staff reports of harm

Spearman’s rho revealed a moderate association between staff- and patient-reported harm of mutually identified events (r_s_ = 0.42, p < 0.001) (near-misses were included as ‘no harm’). patients reported higher harm than staff in 266 (59.6%) events. All events reported as major harm or death by staff were also reported as such by the patient. In contrast, of all 78 mutually identified events reported by patients as resulting in major harm or death, 69.2% were reported by staff as a no harm, low harm, or a near miss.

Specifically, patients reported the following harm levels: near (12.3%), no harm (5.8%), low (32.1%), moderate (26.7%), major (12.6%), and catastrophic (4.9%). Twenty-five events (5.6%) did not include a (sufficient) information on harm. Staff reported: near-miss (7.8%), no harm (50.9%), low (29.8%), moderate (8.5%), major (1.1%), and catastrophic (1.8%).

Examples of low-staff, high-patient reported harm included: kidney failure, organ perforation, permanent disability and death. In such cases, patients described longer-term harm or attributed poor outcomes to hospital failure where staff did not.

### Patient versus staff reports of adjacent events

Patients reported a total of 582 adjacent events in linked complaint narratives (i.e. missed by staff), in contrast to only 127 adjacent events reported by staff across the same time period (i.e. missed by patients).

Adjacent events reported only by patients were: safety (18.6%), institutional (18.2%), quality (17.7%), communication (17.2%), respect and rights (13.4%), listening (8.6%) and environment problems (6.4%). Of all adjacent events reported only by patients, 23.5% were described as causing or contributing to significant harm (moderate, major or death).

*Safety* and *listening* problems were associated with a higher frequency of significant harm as reported in complaints (p < 0.001). Safety problems often included clinical omissions: ‘I was discharged without a scan’. ‘they forgot to administer my required dosage’. Listening problems included missed warning signals (e.g. occurring before a mutually identified event): ‘his screams of pain were ignored’, ‘my symptoms were dismissed’, ‘no one believed me’.

Adjacent events reported only by staff were mainly: medication (23.6%), diagnostics (11.8%), implementation of care (10.2%), pressure ulcers (10.2%), patient falls (9.4%), and infection control (7.1%). Of all adjacent events reported only by staff, only 0.8% were described as resulting in significant harm.

Mosaic plots presenting the association between problem type and harm of adjacent events reported only by patients, or only by staff, can be found in Online Supplement 2.

### Patient versus staff reports of mutually identified events

Patients and staff generally reported the same problem domains (n = 391, 87.7%) underlying mutually identified events. These were *clinical* (n = 260, 58.3%), *management* (n = 106, 23.8%), and rarely *relationship* (n = 25, 5.6%). Problem domains of patient and staff reports differed in 55 (12.3%) cases - for example, where a staff strictly reports a missed diagnosis (*clinical*) while the patient describes that the doctor dismissed their symptoms (*relationship*).

Yet, comparative analysis of the free-text descriptions of these events revealed that even when describing the same overall problem domains, patient and staff provide different explanations as to how these problems occurred. Such differences could largely be divided into *complementary accounts* (n = 115, 25.8% of mutually identified events) and *contested stories* (n = 204, 45.7% of mutually identified events).

#### Complementary accounts

Out of 446 mutually identified events, 115 (25.8%) generated complementary patient and staff accounts (i.e. combining towards a complete explanation of an event). Complementary accounts typically described *protracted events*: events that originate from an earlier, unnoticed error or omission (e.g. failed communication, coordination breakdowns between departments). In these cases, patients typically described consequences of difficult-to-identify failures on their overall care pathway, while staff reports retrospectively examined and described necessary detail on how a care omission or error had occurred.

For example, in one case the patient wrote ‘although my scan was taken two months ago, I only received the diagnosis yesterday’ while staff wrote ‘reading through the patient notes, it appears the scan showed a mass, but no alert was sent to the urologist at the time’.

Corresponding with this, patients were the first to detect the failure in 44.3% of complementary accounts, often through identifying breakdowns in their care journey (i.e. patient-privileged information). For example, patient phrases included: ‘Dr X did not inform Dr Y of test results’, ‘they incorrectly wrote to my GP that I had received the test’, ‘a second opinion confirmed that the diagnosis had been missed’.

In comparison, staff reports revealed retrospective information on how such breakdowns were originally caused internally (e.g. incorrect documentation. failed internal coordination) (i.e. staff-privileged information). Example staff phrases include: ‘it appears no biopsy material was sent to the lab’, ‘the wrong medication was received from the pharmacy’, ‘error in reporting of test results on a previous visit’.

Overall, patients typically provided complementary insights in events reported by staff as problems with *implementation of care* (n = 39, 33.9% of complementary accounts), *diagnostics and investigations* (n = 27, 23.5%), *delayed operations or procedures* (n = 14, 12.1%) and *diagnosis* (n = 12, 10.4%). Patient reports with complementary insights were categorized as *institutional processes* (n = 24, 36.5% of complementary accounts), *safety* (n = 40, 34.8%) and *quality* of care (n = 18, 15.7%) events. Staff reported significant harm in 9 (8.0%) events while patients reported significant harm in 42 (37.2%) events. Differences were typically due to patients providing a longer-term account of harm – for example, progressed cancer due to a significant delay.

#### Contested stories

Of 446 mutually identified events, 204 events (45.7%) generated contested insights on why problems occurred. Contested insights were primarily identified when reports discussed co-witnessed failures (e.g. patient falls, labour incidents), *tightly coupled* errors,^24^ events where negative outcome are clearly linked to an error (e.g. medication error, patient wounds), or significant but unexplained harm (e.g. surgical complications, failure to rescue).

In such cases, patients tended to ascribe responsibility to either staff or the wider hospital while staff reports appeared to minimize or externalize blame. For example, when describing labour incidents, staff reported poor outcomes without referring to aspects of care delivery that may have caused such outcomes: e.g. ‘unexplained stillbirth’, ‘3^rd^-degree tear’. In contrast, patients provided detailed accounts of staff behaviour, clinical decision making and social interactions: e.g. ‘the senior doctor had to take over the delivery after three failed times’, ‘I felt like I was forced to consent’.

In some cases (e.g. often in cases of patient falls), patients and staff appeared to blame each other. For example, staff wrote: ‘patient kept walking around and fell after several attempts to put him back in bed’, while the patient wrote: ‘the nurse refused to help me go to the toilet’.

In cases where harm was evident but unexplained (e.g. surgical complications, failure to rescue), patients implied hospital or staff liability, and demanded a deeper investigation: e.g. ‘I just need to know what happened’, ‘I still have not had any answers’. While staff tended to minimize failures, representing poor outcomes as inherent to clinical uncertainty.^25^ For example, staff wrote ‘unexpected return to the theatre for small obstruction, a simple revision was performed, good post-operative recovery’, while the patient ‘I was failed by my surgeon’.

Overall, contested insights in mutually identified events were typically identified in events reported by staff as problems with *labour and delivery* (n = 54, 26.5% of contested reports), *patient falls* (n = 50, 24.5%), *medication* (n = 37, 18.1%) and *surgical complications* (n = 31, 15.2%). Patient reports were mostly categorized as *safety* (n = 127, 62.2% of contested reports) and *quality* of care (n = 42, 20.6%) events. Events with contesting accounts involved relatively high levels of harm: staff reported significant harm in 36 cases versus patients in 122 cases. This typically resulted from contrasting views on whether harm could be attributed to errors or omissions – for example, harm due to inherent risk (staff) versus due to surgical error (patient).

## Discussion

Incidents where complaints and incident reports overlapped represented a small but critical area of investigation, with disproportionately severe or high-harm incidents. When reporting on mutually identified events, patients often described higher harm than staff, included adjacent events not captured by staff, and provided complementary or contested details on the causes of unsafe care. Our findings reinforce the growing recognition that patient perspectives can contribute to the traditionally staff-driven lens on patient safety,^21^ and call for greater patient involvement in incident monitoring and investigation. Our findings have several implications for research and practice.

First, while the overlap was small, linked complaints and incident reports represented critical incidents (e.g. Serious Incidents, high levels of harm). Moreover, overlapping reports often reflected a point of rupture in patient and staff perspectives on the causes of harm (‘*contested stories*’). The high-severity and disputed nature of this overlap suggests the potential of using patient-level data linkage of complaints and incident reports to identify critical incidents where patient involvement is needed. Identifying overlaps could further help distinguish low-frequency - but serious - incidents amongst a wealth of more mundane incident reports (which comprise the majority of incident reporting systems).^26^

Second, corresponding with systems and human factors approaches to incident analysis,^4,27^ our findings demonstrate that the linkage of safety events to complaints offers the opportunity of gathering occurrences preceding or following mutually identified events that are not reported within incident reporting systems, but are captured in the narrative style of complaints. This includes ‘softer’ or less visible aspects of unsafe care which taxonomies in incident reporting systems are not necessarily set out to capture. In particular, our findings demonstrate that linked complaints can reveal listening failures (i.e. ignored warning signals, patient dismissal) and clinical omissions preceding high-harm clinical events which do not tend to be captured in the incident reporting system. This illustrates how patient-reported narratives can complement the predefined and relatively clinically-focused content of staff incident reports to provide greater insight into the web of (potentially) inter-related problems that contribute to harm.

Third, our findings demonstrate that even when patients and staff identify the same problem themes, narrative explanations of why these problems occur can still differ and both be relevant to learning. This notion is not always reflected in quality improvement literature, which tends to measure the value of free-text datasets by their ability to identify unique themes (relative to existing data repositories) (e.g. Levtzion-Korach et al).^28^ Contrarily, our study shows the value of triangulating different datasets that capture the same occurrences, but from a different angle. Taking notice of discrepancies between patient and staff stories of unsafe care is critical as it can indicate the extent to which the patients’ experiences and needs have been understood.

Fourth, given that nearly three-quarters of patient reports of significant harm (moderate, major or death) were reported as no or low harm by staff, our results call for further research into patient-reported harm as an additional safety monitoring mechanism. Although this might increase the rate of false positives, it would reduce the risk of leaving critical incidents unchecked. An external measure of harm, from a reporter who is independent from a hospital’s safety culture, would further help address the paradox that incident-reporting systems might be least effective in the settings where they are most needed (e.g. due to poor culture and fear or blame), as vividly demonstrated in recent safety scandals.

## Limitations

There are four main limitations in our study. First, problem categories of staff reports included in this study were directly extracted from staff reports, rather than coded by the researchers. Although this aligned with the aim of this study (i.e. which examined reporting behaviour rather than event epidemiology), classifications of staff reports may not always be reliable or accurately reflect the content within free-text descriptions.

Second, it is unclear to what extent adjacent events were actually directly related. As we conceptualized ‘adjacent events’ as any additional event occurring within the time period as described in the complaint, it is expected that the number of patient-reported adjacent events was somewhat higher than staff-reported adjacent events. Further, in line with our argument for linking datasets, the different time-points for reporting between complaints and incident reports will have influenced perceived causes for incidents. Nevertheless, this approach provides the opportunity to increasingly map all the potential factors that might be relevant to safety, and addresses the much-needed data gap on the pathways to harm (i.e. over time and across settings).

Third, some incidents may originally have been reported as higher or lower harm by staff. In English health care settings, the initially reported level of harm may be adjusted by the hospital if a subsequent investigation suggests a different degree of harm. Since all incidents were extracted at least two months after reporting dates, data included in our study therefore represents final harm according to the hospital. On the other hand, this may be considered more comparable to harm reported in complaints, which are submitted retrospectively.

Fourth, in line with the aims and theoretical rationale, this study relies on each reporter’s perception of an incident. This means it is unclear which report provides a more ‘accurate’ depiction of past events. Although some may regard this as a limitation, we have argued that incidents are inherently social and multi-faceted, and that their interpretation will always rely to some degree on a particular viewpoint and socio-cultural context. Patient and staff insights therefore require triangulation for comprehensive safety analysis.

## Conclusion

To achieve a comprehensive understanding of the many factors that may contribute to harm, and to identify potential informational gaps in individual learning systems, it is critical to link and combine insights gained through different reporting systems, generated by heterogeneous stakeholders. This study has demonstrated the value of using patient complaints to supplement, test, and challenge staff reports, providing insight on adjacent problems in their care pathway, and providing differential, and often contested, narratives of why problems occurred. Accordingly, we propose that a more holistic analysis of safety incidents can be achieved through better integration of patient and staff insights on unsafe care, such as through increased data linkage and integration of their respective reporting datasets.

## Supplemental Material

sj-pdf-1-hsr-10.1177_13558196211029323 - Supplemental material for Getting the whole story: Integrating patient complaints and staff reports of unsafe care Click here for additional data file. Supplemental material, sj-pdf-1-hsr-10.1177_13558196211029323 for Getting the whole story: Integrating patient complaints and staff reports of unsafe care by Jackie Van Dael, Alex Gillespie, Tom Reader, Katelyn Smalley, Dimitri Papadimitriou, Ben Glampson, Daniel Marshall and Erik Mayer in Journal of Health Services Research & Policy

sj-pdf-2-hsr-10.1177_13558196211029323 - Supplemental material for Getting the whole story: Integrating patient complaints and staff reports of unsafe care Click here for additional data file. Supplemental material, sj-pdf-2-hsr-10.1177_13558196211029323 for Getting the whole story: Integrating patient complaints and staff reports of unsafe care by Jackie Van Dael, Alex Gillespie, Tom Reader, Katelyn Smalley, Dimitri Papadimitriou, Ben Glampson, Daniel Marshall and Erik Mayer in Journal of Health Services Research & Policy
