# Supplementary material for: Getting the whole story: Integrating patient complaints and staff reports of unsafe care
Source: J Health Serv Res Policy. 2021 Jul 7;27(1):41–9. doi: 10.1177/13558196211029323 (PMC8772011; doi:10.1177/13558196211029323)
Supplement: sj-pdf-1-hsr-10.1177_13558196211029323 - Supplemental material for Getting the whole story: Integrating patient complaints and staff reports of unsafe care [file sj-pdf-1-hsr-10.1177_13558196211029323.pdf]

| | |
|---|---|
| **Article Title:** | **Getting the whole story: integrating patient complaints and staff reports of unsafe care** |
| **Article Authors:** | **van Dael, Jackie; Gillespie, Alex; Reader, Tom; Smalley, Katelyn; Papadimitriou, Dimitri; Glampson, Ben; Marshall, Daniel; Mayer, Erik** |

## Supplement 1: Description of linked data-sets

### Data linkage and preparation

First, we searched for patients with both complaint(s) and staff incident report(s) ("PSIs") in the hospital data-system. For all complaints received between April 2014 and March 2019 (n=5,265), PSI records were searched using patient identifiers to identify patients with at least one complaint and one PSI. Linkage was based on Medical Records Number (MRN), and full name to include records with incorrect or missing MRNs and vice versa.

Second, we checked if linked complaints and PSIs were reported in similar time periods. PSIs reported more than 365 days before, or more than 90 days after, the receipt of a complaint were not included. We chose these cut-offs as complaints can only be lodged up to 365 days after the events have occurred and hospitals are required to investigate a complaint within three months.

Third, we manually reviewed the linked complaints and PSIs that met the time criteria to identify mutually identified events. A total of 909 complaints and 1,594 PSIs linked records were reviewed. Event matching was conducted through manual review of free-text descriptions in PSIs and complaints; supported by data on event date and location (i.e., site; division; ward). Complaints were included if they reported at least one event also identified in PSIs. The robustness of this process was tested with a second rater reliability test.

Finally, incident descriptions in all linked complaints and PSIs with at least one mutually identified event were extracted from the data-system for codification. In addition to descriptions of mutually identified events, all adjacent patient- and staff reported events (i.e., events reported by only patients or only staff in the care period described in the complaint), were extracted and coded.

### Descriptive results

**Table S1** Descriptive details of patient- and staff descriptions of events in our linked study sample

| | **Staff-reported event** | **Patient-reported event** |
|---|---|---|
| Word count (mean) | 61.2 (SD = 45.4) | 96.1 (SD = 63.7)[1] |
| Reporting lapse (in days) | Mean = 13.1 (SD=43.2)<br>Median = 1 | Mean = 70.0 (SD = 91.0)<br>Median = 36 |

[1]Based on key sentences to describe an event; i.e. excluding any other references to the event, or its consequences, throughout the complaint letter.

**Table S2** Nature and severity of Patient Safety Incidents (PSIs) with vs. without an overlapping patient report

| | PSIs without an overlapping patient report (n=81,077) (% in column) | PSIs with an overlapping patient report (n=446) (% in column) | $\chi^2$ | P-value |
|---|---|---|---|---|
| **Category** | | | | |
| Communication | 2404 (3.0) | 17 (3.8) | 1.103 | .294 |
| Diagnosis | 431 (0.5) | 12 (2.7) | 38.256 | <.001 |
| Diagnostics / investigations | 8808 (10.9) | 28 (6.3) | 9.652 | .002 |
| Discharge | 1257 (1.6) | 16 (3.6) | 11.974 | 0.001 |
| Implementation of care | 7518 (9.3) | 40 (9.0) | 0.049 | .825 |
| Infrastructure | 5234 (6.5) | 5 (1.1) | 20.990 | <.001 |
| Labour and delivery | 4624 (5.7) | 54 (12.1) | 33.635 | <.001 |
| Medical device / equipment | 2749 (3.4) | 10 (2.2) | 1.789 | 0.181 |
| Medication | 8893 (11.0) | 37 (8.3) | 3.248 | 0.072 |
| Operations / procedures | 2935 (3.6) | 55 (12.3) | 95.281 | <.001 |
| Patient Information | 2519 (3.1) | 15 (3.4) | 0.097 | 0.756 |
| Pressure ulcer | 3277 (4.1) | 14 (3.1) | 0.933 | .334 |
| Slips, trips, falls | 9566 (11.9) | 50 (11.2) | 0.147 | .701 |
| Transfer | 2421 (3.0) | 9 (2.0) | 1.438 | .231 |
| *Categories <3%* | *15335* | *84* | | |
| | | | | |
| **Serious Incident[1]** | **1139 (1.4)** | **35 (7.8)** | **103.245** | **<.001** |
| *Missing values* | *525 (0.7)* | *4 (0.9)* | | |
| | | | | |
| **Harm** | | | | |
| No | 54290 (67.3) | 227 (50.9) | 54.868 | <.001 |
| Low | 13816 (17.1) | 133 (29.8) | 49.943 | <.001 |
| Moderate | 841 (1.0) | 38 (8.5) | 230.956 | <.001 |
| Major | 55 (0.1) | 5 (1.1) | 66.418 | <.001 |
| Death | 55 (0.1) | 8 (1.8) | 169.911 | <.001 |
| Near-miss | 11045 (13.7) | 35 (7.8) | 12.916 | <.001 |
| *Missing values* | *523 (0.6)* | *0 (0.0)* | | |

[1]A "Serious Incident" is a PSI "where the consequences to patients, families and carers, staff or organisations are so significant or the potential for learning is so great, that a heightened level of response is justified." Source: https://www.england.nhs.uk/wp-content/uploads/2015/04/serious-incidnt-framwrk-upd.pdf.
