# Supplementary material for: Getting the whole story: Integrating patient complaints and staff reports of unsafe care
Source: J Health Serv Res Policy. 2021 Jul 7;27(1):41–9. doi: 10.1177/13558196211029323 (PMC8772011; doi:10.1177/13558196211029323)
Supplement: sj-pdf-2-hsr-10.1177_13558196211029323 - Supplemental material for Getting the whole story: Integrating patient complaints and staff reports of unsafe care [file sj-pdf-2-hsr-10.1177_13558196211029323.pdf]

Article Title:	Getting the whole story: integrating patient complaints and staff reports of unsafe care
Article Authors:	van Dael, Jackie; Gillespie, Alex; Reader, Tom; Smalley, Katelyn; Papadimitriou, Dimitri; Glampson, Ben; Marshall, Daniel; Mayer, Erik

Supplement 2: Mosaic plots

A mosaic plot is a method for visualizing the association between two or more qualitative variables. Mosaic plots graphically represent the conditional relative frequency for a cell in a contingency table and the chi-square test residuals. The size of each tile is proportional to the number of cases in the cell. The colour of each tile reflects the deviation from the expected frequency (residual) from the X^2 test.

Figure S1 the association between problem type and harm in adjacent incidents only reported by patients (n=582)

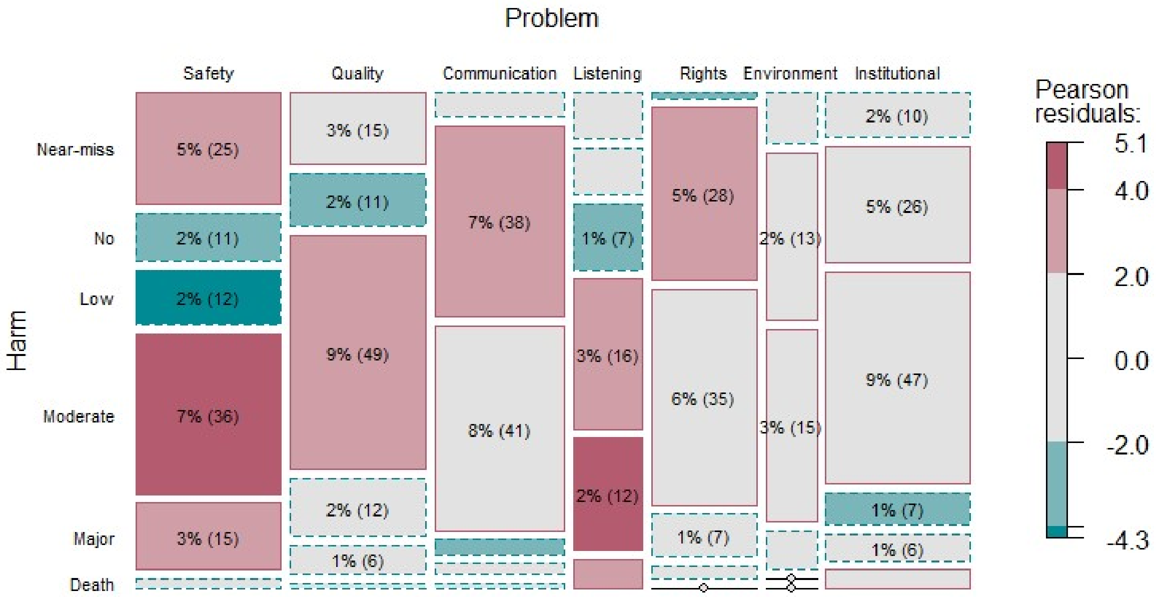

Footnote. The chi-squared test revealed statistically significant patterns: $\chi^2 = 197.4$, df = 30, $p < .001$. Forty-three patient-reported adjacent events reported did not provide sufficient information for the classification of harm and were excluded from the mosaic plot and chi-square test.

Figure S2 the association between problem type and harm in adjacent incidents only reported by staff (n=127)

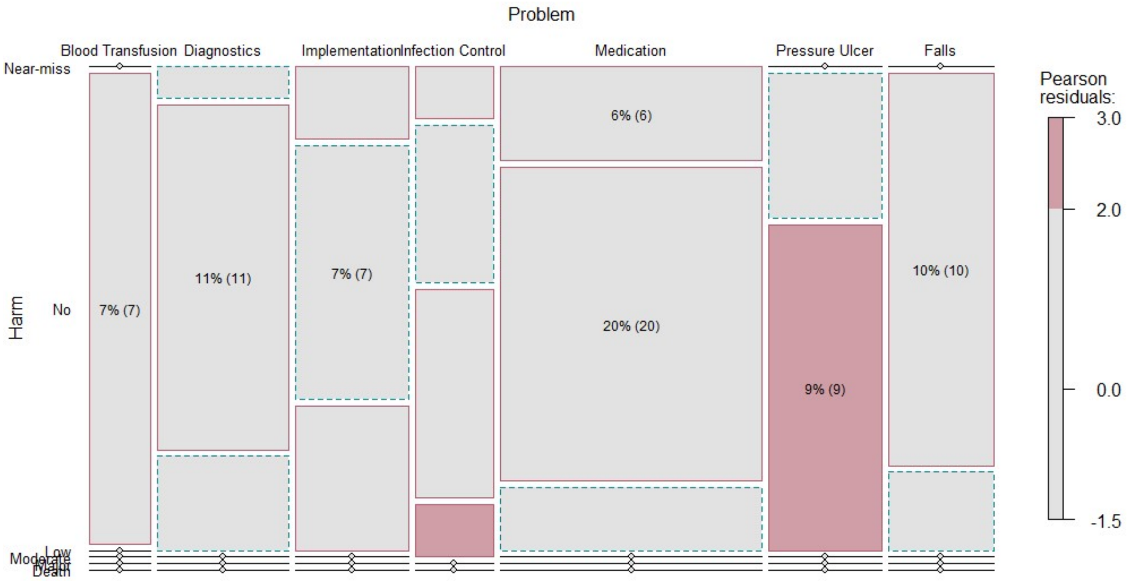

Footnote. All categories with a frequency of lower than 5 were not included in this plot. Count of individual cells in the contingency table were insufficient to conduct a reliable chi-square test.
